# Views, knowledge, and practices of hospital pharmacists about using clinical pharmacokinetics to optimize pharmaceutical care services: a cross-sectional study

**DOI:** 10.1186/s12913-022-07819-4

**Published:** 2022-03-28

**Authors:** Ramzi Shawahna, Naser Shraim, Rafeef Aqel

**Affiliations:** 1 Department of Physiology, Pharmacology and Toxicology, Faculty of Medicine and Health Sciences, An-Najah National University, P.O. Box 7, New Campus, Building: 19, Office: 1340, Nablus, Palestine; 2 An-Najah BioSciences Unit, Centre for Poisons Control, Chemical and Biological Analyses, An-Najah National University, Nablus, Palestine; 3 Department of Pharmacy, Faculty of Medicine and Health Sciences, An-Najah National University, Nablus, Palestine; 4 Master of Clinical Pharmacy Program, Faculty of Graduate Studies, An-Najah National University, Nablus, Palestine

**Keywords:** Education, Curriculum, Knowledge, Pharmacokinetics, Pharmacists, Palestine

## Abstract

**Background:**

Pharmacokinetics (PK) is often used to optimize individualized dosing regimens of some drugs. This study was conducted to determine views, knowledge, and practices of hospital pharmacists in Palestine about using clinical PK to optimize pharmaceutical care services.

**Method:**

This study was conducted in a cross-sectional design using a questionnaire. The questionnaire collected information about the nature of PK courses taught to hospital pharmacists, importance, relevance, effectiveness, adequacy, and depth of these courses, adequacy of PK skills, implementing PK knowledge/skills in current practice, and barriers limiting the implementation of PK to optimize pharmaceutical care services for hospitalized patients. The hospital pharmacists were visited in their places of work and were asked to complete the questionnaire in privacy. Categorical data were compared using Kruskal-Wallis test or Mann-Whitney *U* tests.

**Results:**

The questionnaire was completed by 145 hospital pharmacists. Of the pharmacists, 84 (57.9%) received basic PK courses, 79 (54.5%) were taught integrated PK courses, and 94 (64.8%) agreed that the PK courses were important to their current practice. Similarly, 85 (57.9%) of the pharmacists were not satisfied with the teaching methods and contents of the PK courses. Pharmacists who obtained their degrees from Palestine were less satisfied with the methods of teaching compared to those who obtained their degrees from foreign countries (*p*-value = 0.006). Only 25 (17.2%) pharmacists reported frequent use of PK knowledge in their current practice. Lack of practical knowledge and continuing education, poor understanding of PK by pharmacists and other healthcare professionals were identified as barriers limiting the implementation of PK in optimizing pharmaceutical care services to hospitalized patients.

**Conclusion:**

The hospital pharmacists were generally not satisfied with the way PK courses were taught and expressed difficulty in implementing PK knowledge and skills to improve pharmaceutical care services to hospitalized patients. Integrating PK topics within other relevant courses and adopting more clinically oriented learning methods could improve understanding and implementing PK knowledge and skills in optimizing pharmaceutical services to hospitalized patients. Further studies are still needed to determine the optimal teaching/learning methods that can improve knowledge and skill acquisitions of pharmacists in the area of PK.

**Supplementary Information:**

The online version contains supplementary material available at 10.1186/s12913-022-07819-4.

## Background

Pharmacokinetics (PK) is one of the tools that can be used to optimize pharmaceutical care services to hospitalized patients [1]. The principles of PK are often applied in modeling concentrations of drugs to find optimal dosage, determine exposure to drugs, and understand the disposition of drugs within the human body. In clinical practice, healthcare providers apply the principles of PK to design/optimize individualized doses of some drugs, monitor drug concentrations, maximize the intended therapeutic outcomes and minimize toxicities [2–5]. It is well-established that implementing the principles of PK can help achieve optimal therapeutic drug concentrations, reduced patient mortality/morbidity, reduce costs associated with therapy, and improve the overall health outcomes of the patients [5–8].

PK courses were listed as required didactic elements by the Accreditation Council for Pharmacy Education (ACPE) in the Doctor of Pharmacy (PharmD) degree program [9]. Today, different pharmacy schools around the world offer standalone courses in PK [3]. Alternatively, PK topics are integrated within pharmacology, pharmaceutics/biopharmaceutics, and/or other relevant courses. In their accreditation standards report, the ACPE suggested teaching topics related to the applications of PK in optimizing drug delivery, calculations of individualized drug doses, monitoring drug concentrations in different biological fluids/matrices, and adjusting drug doses as appropriate to achieve therapeutic drug concentrations [9].

In modern healthcare systems, multi-healthcare provider team approaches are increasingly used in providing care to hospitalized patients [10–12]. As a result, hospital pharmacists are increasingly integrated as direct healthcare providers in these teams. Applying the principles of PK is one of the main responsibilities of hospital pharmacists who provide pharmaceutical care services to hospitalized patients [3]. Pharmacists are supposed to use their PK knowledge and skills to make informed decisions and/or recommendations to improve the outcomes of the patients. Applying the principles of PK requires an adequate understanding of the processes of absorption, distribution, metabolism, and excretion of drugs in certain patient populations and pathological conditions. Previous studies have reported that pharmacy students and pharmacists described PK courses and applying the principles of PK in clinical practice as difficult and challenging [3, 13–17]. Additionally, pharmacy students reported poor evaluation and low engagement in PK courses offered by pharmacy schools [14, 15, 17]. Probably, this could be attributed to the mathematical nature of the PK courses, failure of pharmacy students to realize the importance of PK to their future roles, lack of sufficient education/training and acquisition of skills in PK, and lack of confidence of pharmacists in applying their knowledge and skills [3, 13–17].

Lately, pharmacy practice has undergone tremendous advancements in different countries around the world. Consequently, pharmacists are assuming more roles in providing direct and indirect patient care services [10]. Today, many hospitals offer therapeutic drug monitoring and other PK-related care services for inpatients and outpatients [5, 18, 19]. Currently, little is known about the views, knowledge, and practices of Palestinian hospital pharmacists about using the principles of clinical PK to optimize pharmaceutical care services. Additionally, little is known about how hospital pharmacists perceive their education/training in PK. Therefore, this study was conducted to determine views, knowledge, and practices of hospital pharmacists about using clinical PK to optimize pharmaceutical services and outcomes of hospitalized patients.

## Methods

### Study design and settings

This study was conducted in a cross-sectional design. A questionnaire was used to collect the data from pharmacists practicing in different Palestinian hospitals. The data collection was conducted in the period between August 2018 and November 2018. This study was conducted in adherence to the strengthening the reporting of observational studies in epidemiology (STROBE) statement [20]. Adherence to the STROBE statement is shown in Supplementary Table S1.

### Study population and sampling procedure

The study population in this study was all pharmacists practicing in Palestinian hospitals across the West Bank of Palestine. According to the Palestinian Health Information Center, 178 pharmacists were employed by Palestinian hospitals across the West Bank at the time of the study. The sample size needed for this study was computed using an online software program (Raosoft sample size calculator: (http://www.raosoft.com/samplesize.html). The sample size was computed at a 95% confidence interval and a pre-set margin of error of 5%. The sample size needed for this study was 122 pharmacists. To ensure recruiting the sample size needed in this study, all pharmacists employed by Palestinian hospitals across the West Bank at the time of the study were invited to take part in the study. In this study, licensed pharmacists who were practicing in a hospital in the West Bank of Palestine were recruited. Pharmacists who practiced in community settings and those employed in the pharmaceutical industry or any other sector were excluded.

The pharmacists were visited in their places of work by one researcher (RA) who invited them to participate in the study and obtained their informed consent. The pharmacists responded to the questionnaire in privacy.

### The questionnaire and data collection

The questionnaire used in this study was developed after a review of the relevant literature [3, 21–27]. The questionnaire contained 7 sections (Supplementary Table S2). The first section collected the demographic and professional characteristics of the hospital pharmacists. The second section collected information on the nature of PK courses that hospital pharmacists received during their pharmacy education and how these courses were taught. The third section contained 6 statements about the importance, relevance, effectiveness, adequacy, and depth of the PK courses that the hospital pharmacists received during their education. The pharmacists were asked to rate these statements using a Likert scale of 1–5 (1 = strong disagreement, 5 = strong agreement). The fourth section contained a statement about the use of PK knowledge in current practice. The hospital pharmacists were asked to rate this statement using a Likert scale of 1–5 (1 = none of the time, 5 = all/most of the time). The fifth section contained a statement on the adequacy of PK skills. The hospital pharmacists were asked to rate this statement using a Likert scale of 1–5 (1 = complete inadequacy, 5 = complete adequacy). The sixth section contained a statement about the difficulty of implementing PK knowledge and skills. The hospital pharmacists were asked to rate this statement using a scale of 1–10 (1 = low difficulty, 10 = high difficulty). Finally, the seventh section contained 5 statements about the potential barriers that limit the application of clinical PK to optimize pharmaceutical care services and outcomes of the patients. The hospital pharmacists were asked to rate this statement using a Likert scale of 1–5 (1 = extremely unimportant, 5 = extremely important).

The questionnaire used in this study was pilot tested with 10 pharmacists who did not participate in the larger study to ensure that the questionnaire was readable and easily comprehensible. Based on the feedback of the pharmacists, some statements were rephrased for clarity. To ensure the questionnaire was reliable, the 10 pharmacists were asked to respond to the questionnaire twice. A time period of 30 min to 2 h was let between both administrations. The test-retest reliability was used to ensure the stability of the score over a short time. Scores of the 10 pharmacists obtained in the two rounds were correlated using Pearson’s correlations. The Pearson’s r was 0.93, *p*-value < 0.001 which indicated stable scores and a reliable questionnaire. The internal consistency was tested using Cronbach’s alpha statistics. The sections that contained more than 1 item (section 3 and section 7) had a Cronbach’s alpha of > 0.70 which indicated acceptable internal consistency.

### Data analysis

The data obtained in this study were analyzed using the IBM Statistical Package for Social Sciences version 21 (SPSS v.21). Kolmogorov-Smirnov test was used to assess whether the data followed normal distribution or not. As the data did not follow a normal distribution, non-parametric tests were used to analyze the data. The data were expressed using medians with their corresponding lower and upper quartiles [Q1, Q3]. Categorical data were compared using the Kruskal-Wallis test or Mann-Whitney *U* test as appropriate. *P*-values < 0.05 were considered statistically significant.

### Ethics approval and consent to participate

This study was conducted in adherence to the international ethical guidelines and those in the Declaration of Helsinki. This study belonged to the “Exempt” review category as it involved no/minimal risk to the participants. This exemption was approved by the Institutional Review Board (IRB) of An-Najah National University (An-Najah IRB#: 29-Apr-18), the Medical Research Committee of the respective hospitals, and by the Health Education Office of the Ministry of Health (Protocol #: 162/866/18). Therefore, verbal informed consent was required for this study. The IRB approved this verbal consent. The data collected in this study were analyzed anonymously.

## Results

### Demographic and clinical characteristics of the pharmacists

Of the 178 originally invited hospital pharmacists, 145 completed the questionnaire; giving a response rate of 81.5%. The majority of the hospital pharmacists were female (78.6%), younger than 40 years old (79.3%), had a (BSc) degree in pharmacy (63.4%), obtained their first pharmacy degree less than 10 years ago (64.1%), and were employed by private hospitals (52.4%). Of the hospital pharmacists, 71.7% obtained their pharmacy degree from a university in Palestine. The other countries from where the hospital pharmacists obtained their pharmacy degree included: Jordan, the Russian Federation, Italy, Syria, Iraq, Greece, France, Egypt, United Arab Emirates, the Philippines, Australia, Pakistan, and Turkey (Supplementary Table S3). The detailed demographic and professional characteristics of the hospital pharmacists are shown in Table 1.

**Table 1:** Demographic and professional characteristics of the hospital pharmacists (*n = 145*)

Characteristic	n (%)
**Gender**	
Male	31 (21.4%)
Female	114 (78.6%)
**Age (years)**	
< 40	115 (79.3%)
≥ 40	30 (20.7%)
**Academic degree in pharmacy**	
BSc Pharmacy	92 (63.4%)
PharmD	28 (19.3%)
MSc/PhD in Pharmacy	25 (17.2%)
**Country from where the pharmacy degree was obtained**	
Palestine	104 (71.7%)
Other	41 (28.3%)
**Time elapsed since the pharmacy degree was obtained (years)**	
< 10	93 (64.1%)
≥ 10	52 (35.9%)
**Length of working experience as a pharmacist in a hospital (years)**	
< 5	69 (47.6%)
≥ 5	76 (52.4%)
**Type of hospital**	
Governmental	68 (46.9%)
Private	77 (53.1%)

*BSc* Bachelor of Science, *MSc* Master of Science, *PharmD* Doctor of Pharmacy, *PhD* Doctor of Philosophy

### Nature of the PK courses and how these courses were taught

Of the hospital pharmacists, 84 (57.9%) stated that they have received basic PK courses during their pharmacy education. Of the hospital pharmacists, 79 (54.5%) stated that the PK courses were integrated/part of other courses like pharmacotherapy, pharmaceutics, and/or pharmacology. In this study, only 17 (11.7%) hospital pharmacists stated that they have received continuing education related to PK. Detailed answers of the hospital pharmacists are shown in Table 2.

**Table 2:** Nature of PK courses and how these courses were taught

Item	n (%)
**Nature of PK courses**	
Basic	84 (57.9%)
Clinical	9 (6.2%)
Both	52 (35.9%)
**How PK courses were taught**	
Standalone courses	66 (45.5%)
Integrated/part of other courses like pharmacotherapy, pharmaceutics, and/or pharmacology	79 (54.5%)
**Received continuing education courses related to PK**	
Yes	17 (11.7%)
No	128 (88.3%)

*PK* Pharmacokinetics

### Views of the hospital pharmacists on the PK courses received during their pharmacy education

Views of the hospital pharmacists on the importance, relevance, effectiveness, adequacy, and depth of the PK courses received during their pharmacy education are shown in Table 3.

**Table 3:** Views of the hospital pharmacists on the importance, relevance, effectiveness, adequacy, and depth of the PK courses received during pharmacy education

		Strongly disagree	Disagree	Neutral	Agree	Strongly agree	
#	Item	n (%)	n (%)	n (%)	n (%)	n (%)	Median [Q1, Q3]
1	The PK courses I received during my pharmacy education were important to my current practice	4 (2.8%)	11 (7.6%)	36 (24.8%)	67 (46.2%)	27 (18.6%)	4 [3, 4]
2	The PK courses I received during my pharmacy education were relevant to my current practice	4 (2.8%)	15 (10.3%)	42 (29.0%)	69 (47.6%)	15 (10.3%)	4 [3, 4]
3	The PK courses I received during my pharmacy education could have been taught in a better way	2 (1.4%)	9 (6.2%)	49 (33.8%)	66 (45.5%)	19 (13.1%)	4 [3, 4]
4	The method used to teach the PK courses during my pharmacy education were effective	6 (4.1%)	19 (13.1%)	55 (37.9%)	57 (39.3%)	8 (5.5%)	3 [3, 4]
5	The contents of the PK courses I received during my pharmacy education were adequate	6 (4.1%)	31 (21.4%)	48 (33.1%)	57 (39.3%)	3 (2.1%)	3 [2, 4]
6	The depth of the PK courses I received during my pharmacy education was appropriate to prepare me for my future clinical roles	7 (4.8%)	33 (22.8%)	51 (35.2%)	47 (32.4%)	7 (4.8%)	3 [2, 4]

*PK* pharmacokinetics, *Q1* lower quartile, *Q3* upper quartile

Of the hospital pharmacists, 94 (64.8%) agreed that the PK courses they received during their pharmacy education were important to their current practice. The hospital pharmacists who had MSc/PhD degrees tended to express more agreement compared to those who had BSc or PharmD degrees (median scores: 4 [4, 5] vs. 4 [3, 4], *p*-value = 0.006). Detailed scores are shown in Supplementary Table S4.

Of the hospital pharmacists, 84 (57.9%) agreed that the PK courses they received during their pharmacy education were relevant to their current practice. The hospital pharmacists who were male and those who obtained their pharmacy degree from another country tended to express more agreements compared to those who were female and those who obtained their pharmacy degree from Palestine (median scores: 4 [3, 4] vs. 3 [3, 4], *p*-value = 0.015 and 4 [3, 4] vs. 3 [3, 4], *p*-value = 0.006, respectively) (Supplementary Table S4).

Of the hospital pharmacists, 85 (58.6%) stated that the PK courses they received during their pharmacy education could have been taught in a better way. The hospital pharmacists who obtained their pharmacy degrees from Palestine tended to express more agreement compared to those who obtained their pharmacy degrees from another country (median scores: 4 [3, 4] vs. 3 [3, 4], *p*-value = 0.001) (Supplementary Table S4).

Consistently, 80 (55.2%) hospital pharmacists did not agree that the methods used to teach the PK courses during their pharmacy education were effective. The hospital pharmacists who obtained their pharmacy degrees from another country tended to express more agreement compared to those who obtained their pharmacy degree from Palestine (median scores: 4 [3, 4] vs. 3 [3, 4], *p*-value = 0.006) (Supplementary Table S4).

Again, 85 (58.6%) hospital pharmacists did not agree that the contents of the PK courses they received during their pharmacy education were adequate. The hospital pharmacists who obtained their pharmacy degrees from another country, worked in private hospitals, and received clinical courses in PK tended to express more agreements compared to those who obtained their pharmacy degree from Palestine, worked in governmental hospitals, and received more basic PK courses (median scores: 4 [3, 4] vs. 3 [2, 4], *p*-value = 0.001, 3 [3, 4] vs. 3 [2, 4], *p*-value = 0.022, 4 [4, 4] vs. 3 [2, 4] and 3 [3, 4], *p*-value = 0.001, respectively) (Supplementary Table S4).

Once more, 91 (62.8%) hospital pharmacists did not agree that the depth of the PK courses they received during their pharmacy education was appropriate to prepare them for their future clinical roles. The hospital pharmacists who were 40 years and older, worked in private hospitals, and received clinical courses in PK tended to express more agreements compared to those who were younger than 40 years old, worked in governmental hospitals, and received more basic PK courses (median scores: 4 [3, 4] vs. 3 [2, 4], *p*-value = 0.042, 3 [3, 4] vs. 3 [2, 3], p-value = 0.013, 3 [3, 4] vs. 3 [2, 4], *p*-value = 0.009, respectively) (Supplementary Table S4).

*PK* Pharmacokinetics, *Q1* lower quartile, *Q3* upper quartile

### Views of the hospital pharmacists on the utilization of PK knowledge in their current practice

Of the hospital pharmacists, only 25 (17.2%) reported frequent utilization of PK knowledge gained through their pharmacy education in their current practice. On the other hand, 53 (36.6%) hospital pharmacists believed that their PK skills adequately allowed them to provide optimal patient care. The hospital pharmacists who received integrated PK courses and those who received their pharmacy degree from another country tended to express more agreements compared to the pharmacists who received standalone courses and those who obtained their pharmacy degree from Palestine (median scores: 4 [4, 5] vs. 4 [3, 5], *p*-value = 0.027, 5 [4, 5] vs. 4 [3, 5], p-value = 0.040, respectively) (Supplementary Table S4). When the hospital pharmacists were asked to report on a scale of 1–10 (1 being the lowest, and 10 being the highest) the degree to which they thought that PK knowledge and skills were difficult to implement, 108 (74.5%) hospital pharmacists scored 5 and more.

### Barriers limiting the application of PK in optimizing pharmaceutical care services in Palestinian hospitals

Views of the hospital pharmacists on the barriers to the application of PK in optimizing pharmaceutical care services in Palestinian hospitals are shown in Table 4.

**Table 4:** Views of the hospital pharmacists on the barriers limiting the application of PK in optimizing pharmaceutical care services

		Extremely unimportant barrier	Unimportant barrier	Neutral	Important barrier	Extremely important barrier	
#	Item	n (%)	n (%)	n (%)	n (%)	n (%)	Median [Q1, Q3]
1	Lack of practical knowledge	0 (0.0%)	5 (3.4%)	37 (25.5%)	68 (46.9%)	35 (24.1%)	4 [3, 4]
2	Lack of continuing education relevant to PK	1 (0.7%)	7 (4.8%)	27 (18.6%)	80 (55.2%)	30 (20.7%)	4 [4, 4]
3	Lack of role model at work place who knows and applies PK	4 (2.8%)	49 (33.8%)	63 (43.4%)	29 (20.0%)	0 (0.0%)	4 [3, 4]
4	Poor understanding of PK by the health care professionals other than pharmacists	0 (0.0%)	5 (3.4%)	45 (31.0%)	55 (37.9%)	40 (27.6%)	4 [3, 5]
5	Poor understanding of PK by pharmacists	1 (0.7%)	3 (2.1%)	42 (29.0%)	67 (46.2%)	32 (22.1%)	4 [3, 4]

*PK* pharmacokinetics, *Q1* lower quartile, *Q3* upper quartile

Of the hospital pharmacists, 103 (71.0%) rated lack of practical knowledge as an important barrier. The hospital pharmacists who received standalone PK courses tended to rate lack of practical knowledge as an important barrier compared to those who received integrated PK courses (median scores: 4 [4, 5] vs. 4 [3, 4], *p*-value = 0.022) (Supplementary Table S4).

Similarly, 110 (75.9%) hospital pharmacists rated the lack of continuing education relevant to PK as an important barrier. The hospital pharmacists who received standalone PK courses tended to rate lack of continuing education relevant to PK as an important barrier compared to those who received integrated PK courses (median scores: 4 [4, 4] vs. 4 [3, 4], *p*-value = 0.009) (Supplementary Table S4).

Consistently, 95 (65.5%) hospital pharmacists rated poor understanding of PK by the health care professionals other than pharmacists as an important barrier. The hospital pharmacists who received their pharmacy degree from Palestine tended to rate poor understanding of PK by the health care professionals other than pharmacists as an important barrier compared to those who received their pharmacy degree from other countries (median scores: 4 [3, 5] vs. 4 [3, 4], *p*-value = 0.031) (Supplementary Table S4).

Again, 99 (68.3%) hospital pharmacists rated poor understanding of PK by pharmacists as an important barrier. Similarly, pharmacists who were female and those who received their pharmacy degree from Palestine tended to rate poor understanding of PK by pharmacists as an important barrier compared to male pharmacists and those who received their pharmacy degree from other countries (median scores: 4 [3, 4] vs. 4 [3, 4], *p*-value = 0.028, 4 [3, 4.5] vs. 4 [3, 4], *p*-value = 0.025, respectively) (Supplementary Table S4). On the other hand, 29 (20.0%) hospital pharmacists rated lack of role model at the workplace who knew and applied PK as an important barrier.

*PK* Pharmacokinetics, *Q1* lower quartile, *Q3* upper quartile

## Discussion

The use of individualized dosage regimens is becoming increasingly common in hospitalized patient settings [2–5]. Hospital pharmacists often apply the principles of PK to design individualized dosage regimens. This study sought to determine views, knowledge, and practices of hospital pharmacists about using clinical PK in optimizing pharmaceutical care services and outcomes of the patients. To the best of our knowledge, views, knowledge, and practices of Palestinian hospital pharmacists about using clinical PK were not analyzed before. The findings of this study showed that the majority of hospital pharmacists perceived PK knowledge and skills as important and relevant to their practice. On the other hand, the study identified areas of dissatisfaction with the methods used to teach PK courses, contents, and depth of the PK courses to adequately prepare the hospital pharmacists for their future clinical practice. The hospital pharmacists also expressed difficulties in implementing PK knowledge and skills in clinical practice. Barriers limiting the application of PK in optimizing pharmaceutical care services were also identified.

The ACPE recommended teaching applications of PK in optimizing drug delivery, calculations of individualized drug doses, monitoring drug concentrations in different biological fluids/matrices, and adjusting drug doses as appropriate to achieve therapeutic drug concentrations to PharmD students [28]. In clinical practice, pharmacists are supposed to use these principles to optimize individualized drug dosage regimens, maximize the intended therapeutic outcomes, and minimize toxicities [2–5]. Through these healthcare services, hospital pharmacists are expected to reduce mortality/morbidity among hospitalized patients, reduce costs associated with therapy, and improve the overall health outcomes of the patients [5–9]. To provide optimal care services, pharmacists should be equipped with adequate knowledge and skills in PK [3].

Basic, clinical, and a blend of both basic and clinical PK courses are offered by pharmacy schools around the world [3, 29, 30]. In Palestine, pharmacy schools offer two undergraduate degree programs in pharmacy: BSc and PharmD. Additionally, an MSc in clinical pharmacy is also offered. The majority of pharmacy schools offer basic PK courses as required elements in the BSc degree program. On the other hand, more clinically oriented PK courses are offered in PharmD and MSc programs. Because the hospital pharmacists who participated in this study received their pharmacy degrees from different schools in Palestine and elsewhere, they reported receiving basic, clinical, and a blend of both basic and clinical PK courses. The findings of this study showed that the hospital pharmacists who received clinical PK courses were more likely to rate the contents of the PK courses as adequate with appropriate depth to prepare them for their clinical practice. In clinically oriented PK courses, educators could have discussed more applications of PK in real-life scenarios and patient cases that could be encountered in daily practice compared to basic PK courses [14]. In this study, more than half of the hospital pharmacists stated that they have received integrated PK courses. In previous studies, the majority of the pharmacists reported receiving standalone PK courses [3, 26, 29, 31]. Findings of this study showed that the hospital pharmacists who received more integrated courses were more likely to believe that their PK skills adequately allowed them to provide optimal patient care. Additionally, they were more likely to consider lack of practical knowledge of PK and lack of continuing education relevant to PK were important barriers limiting the application of PK in optimizing pharmaceutical care services. Probably, the hospital pharmacists who have received PK courses that were integrated within pharmacotherapy, pharmaceutics, and/or pharmacology were more able to relate PK knowledge and skills that could be used to optimize pharmaceutical care services with other clinical/therapeutic areas. Taken together, these findings might suggest that educators need to consider horizontal and vertical integration of knowledge and skills gained through PK courses within the pharmacy curriculum to enable future pharmacists to relate PK and clinical/therapeutic knowledge that could be used to optimize pharmaceutical care services. It is noteworthy mentioning that the pharmacy curricula offered in different pharmacy schools might contain more than one PK course [3]. The contents of these PK courses might differ significantly [3, 26, 29, 31].

In this study, the majority of the hospital pharmacists agreed on the importance of PK courses they received during their pharmacy education. These findings were consistent with those previously reported among hospital pharmacists in Qatar [3]. Pharmacists are classical experts in medications whose roles include optimizing pharmacotherapy through pharmaceutical care services. Mastery of PK, pharmacodynamics and their application in optimizing pharmacotherapy are core competencies of pharmacists in modern healthcare delivery [32]. Through pharmaceutical care services, pharmacists are supposed to help patients achieve therapeutic drug levels within a lesser number of days, shorten the length of hospitalization, reduce mortality and morbidity, and improve patient outcomes [33, 34]. In this study, the hospital pharmacists who had MSc/PhD in pharmacy were more likely to agree on the importance of PK courses compared to those who had basic pharmacy degrees. Probably, pharmacists with higher academic qualifications assumed managerial/decision-making positions and could be responsible for the provision of more delicate pharmaceutical care services to hospitalized patients. Therefore, those hospital pharmacists are more involved in using PK services including therapeutic drug monitoring. Similarly, the hospital pharmacists who were male and those who obtained their pharmacy degrees from a foreign country were more likely to agree on the relevance of their PK courses to their current practice. Previous studies have shown that male and foreign qualified professionals were more likely to assume managerial/decision-making roles compared to female and nationally qualified professionals [35–37].

The hospital pharmacists in this study were not satisfied with the methods used to teach the PK courses. Foreign qualified pharmacists expressed more agreement on the effectiveness of the teaching methods compared to locally qualified pharmacists. Probably, this could be explained by variabilities in the teaching methods. Team-based learning methods were shown to improve scores in clinical examinations compared to lecture-based learning methods in PK [14]. In this study, more than half of the pharmacists articulated concerns over the adequacy and contents of the PK courses they have received during pharmacy education. The findings of this study were consistent with those reported in previous studies [3, 30, 38]. In Qatar, hospital pharmacists did not agree on the adequacy and depth of the PK courses in preparing them for their current clinical roles [3]. In Pakistan, pharmacists articulated concerns over theoretical/practical imbalance in the pharmacy curricula [30]. It has been argued that adequately planned/designed internships could prepare future pharmacists to assume more clinical roles [38]. In this study, more than half of the hospital pharmacists considered poor understanding of PK by other healthcare professionals as an important barrier limiting the application of PK in optimizing pharmaceutical care services. Many modern healthcare systems around the world have transitioned to multi-healthcare professional team care approaches. In these systems, pharmacists often make important recommendations. Previous studies have shown that recommendations of pharmacists are often accepted by senior prescribers [39]. In the Palestinian healthcare system, pharmacists have recently assumed clinical roles in providing direct pharmaceutical care services to hospitalized patients. This might explain why only a small percentage of the hospital pharmacists were able to apply their PK knowledge in optimizing pharmaceutical care services in this study. In addition to improving the knowledge and skills, of pharmacists, educators and decision-makers should also consider improving understanding of PK by other health care professionals like physicians and nurses. This might improve recognition of the clinical roles of pharmacists in providing direct patient care by the other team members in a multi-healthcare provider approach.

### Strength and limitations of the study

The findings of this study should be interpreted considering several strengths and limitations. First, views, knowledge, and practices of Palestinian hospital pharmacists about using clinical PK to optimize pharmaceutical services and outcomes of patients were collected and analyzed for the first time. Second, a high response rate was obtained in this study. The pharmacists belonged to both genders, were employed by governmental and private hospitals, received their pharmacy degrees from Palestine as well as from other foreign countries, had different academic degrees in pharmacy, and had a variable length of practical experience. This diversity should have ensured the representation of the entire population of pharmacists in Palestine and should have improved the external validity of the findings. Third, although the questionnaire was based on previous studies, the reliability and internal consistency of the questionnaire were assessed in a pilot test. This should have added rigor to the findings of this study. On the other hand, this study had some limitations. First, desirability bias could not be ruled out as participants might have attempted to provide more favorable answers. Second, recall bias could not be ruled out as the pharmacists were asked to express their views on a course that they have taken in the past while they were students. Third, Dunning–Kruger Effect could not be ruled out as pharmacists with poor competency might have overestimated their knowledge and skills in PK [40]. Fourth, the departments in which the hospital pharmacists practiced were not included in the analysis. Pharmacists who practiced in certain departments might have applied PK knowledge more often than other pharmacists.

## Conclusion

Palestinian hospital pharmacists viewed PK courses taken during their pharmacy education as important and relevant to their current practice. The pharmacists were generally not satisfied with the way PK courses were taught and expressed difficulty in applying PK knowledge and skills to improve pharmaceutical care services. Integrating PK topics within other relevant courses and adopting a more clinically oriented learning method could improve understanding and application of PK knowledge and skills. The findings of this study could be informative to decision-makers in academia and professional groups who might need to design interventions to improve knowledge and skills acquisition of PK and their application in optimizing pharmaceutical care services provided to patients in hospitalized patient settings. Further studies are still needed to determine the optimal teaching/learning methods that can improve knowledge and skill acquisitions of pharmacists in the area of PK.

## Supplementary Information

**Additional file 1.**

## Data Availability

All the data relevant to this work are included within the manuscript or provided as supplementary materials. The datasets used and/or analyzed during the current study are available from the corresponding author on reasonable request.
