# Correction to: Views, knowledge, and practices of hospital pharmacists about using clinical pharmacokinetics to optimize pharmaceutical care services: a cross-sectional study

**DOI:** 10.1186/s12913-022-07940-4

**Published:** 2022-04-20

**Authors:** Ramzi Shawahna, Naser Shraim, Rafeef Aqel

**Affiliations:** 1 Department of Physiology, Pharmacology and Toxicology, Faculty of Medicine and Health Sciences, An-Najah National University, P.O. Box 7, New Campus, Building: 19, Ofce: 1340, Nablus, Palestine; 2 An-Najah BioSciences Unit, Centre for Poisons Control, Chemical and Biological Analyses, An-Najah National University, Nablus, Palestine; 3 Department of Pharmacy, Faculty of Medicine and Health Sciences, An-Najah National University, Nablus, Palestine; 4 Master of Clinical Pharmacy Program, Faculty of Graduate Studies, An-Najah National University, Nablus, Palestine

**Correction to: BMC Health Serv Res 22, 411 (2022)**

**https://doi.org/10.1186/s12913-022-07819-4**

Following publication of the original article [1], the authors raised missing corresponding authorship for the second author: both Ramzi Shawahna and Naser Shraim should be listed as corresponding authors, instead of only Ramzi Shawahna.

The author group has been updated above and the original article [1] has been corrected.
