# Supplementary material for: Views, knowledge, and practices of hospital pharmacists about using clinical pharmacokinetics to optimize pharmaceutical care services: a cross-sectional study
Source: BMC Health Serv Res. 2022 Mar 28;22:411. doi: 10.1186/s12913-022-07819-4 (PMC8962057; doi:10.1186/s12913-022-07819-4)
Supplement: Supplementary file 1 — Additional file 1. [file 12913_2022_7819_MOESM1_ESM.docx]

**Supplementary materials for the manuscript:**

**Views, knowledge, and practices of hospital pharmacists with regard to using clinical pharmacokinetics to optimize pharmaceutical care services: A cross-sectional study**

Ramzi Shawahna^1,2*^, Naser Shraim^3^, Rafeef Aqel^4^

^1^Department of Physiology, Pharmacology and Toxicology, Faculty of Medicine and Health Sciences, An-Najah National University, Nablus, Palestine

^2^An-Najah BioSciences Unit, Centre for Poisons Control, Chemical and Biological Analyses, An-Najah National University, Nablus, Palestine

^3^Department of Pharmacy, Faculty of Medicine and Health Sciences, An-Najah National University, Nablus, Palestine

^4^Master of Clinical Pharmacy Program, Faculty of Graduate Studies, An-Najah National University, Nablus, Palestine

**^*^Correspondence:**

Ramzi Shawahna, PhD, Department of Physiology, Pharmacology and Toxicology, Faculty of Medicine & Health Sciences, New Campus, Building: 19, Office: 1340, An-Najah National University, P.O. Box 7, Nablus, Palestine

Phone: + (970) 923 45113 ext 2772

Phone: + (970) 92349739

Email: [ramzi_shawahna@hotmail.com](mailto:ramzi_shawahna@hotmail.com)

**Supplementary Table S1:** Adherence to the strengthening the reporting of observational studies in epidemiology (STROBE) statement [[18](#_ENREF_18)]

|  | Item No | Recommendation | Place in the manuscript |
| --- | --- | --- | --- |
| **Title and abstract** | 1 | (*a*) Indicate the study’s design with a commonly used term in the title or the abstract | Title and abstract |
|  |  | (*b*) Provide in the abstract an informative and balanced summary of what was done and what was found | Abstract adheres to the requirements of the journal |
| Introduction | | | |
| Background/rationale | 2 | Explain the scientific background and rationale for the investigation being reported | Provided in the Background section |
| Objectives | 3 | State specific objectives, including any pre-specified hypotheses | Provided in the last paragraph of the Background section |
| Methods | | | |
| Study design | 4 | Present key elements of study design early in the paper | Provided under Study design and settings |
| Setting | 5 | Describe the setting, locations, and relevant dates, including periods of recruitment, exposure, follow-up, and data collection | Provided under Study design and settings |
| Participants | 6 | (*a*) Give the eligibility criteria, and the sources and methods of selection of participants | Provided under Study population and sampling procedure section |
| Variables | 7 | Clearly define all outcomes, exposures, predictors, potential confounders, and effect modifiers. Give diagnostic criteria, if applicable | Provided under The questionnaire and data collection section |
| Data sources/ measurement | 8* | For each variable of interest, give sources of data and details of methods of assessment (measurement). Describe comparability of assessment methods if there is more than one group | Provided under The questionnaire and data collection section |
| Bias | 9 | Describe any efforts to address potential sources of bias | Provided under The questionnaire and data collection section |
| Study size | 10 | Explain how the study size was arrived at | Provided under Study population and sampling procedure section |
| Quantitative variables | 11 | Explain how quantitative variables were handled in the analyses. If applicable, describe which groupings were chosen and why | Provided under The questionnaire and data collection section |
| Statistical methods | 12 | (*a*) Describe all statistical methods, including those used to control for confounding | Provided under Data analysis section |
|  |  | (*b*) Describe any methods used to examine subgroups and interactions | Provided under Data analysis section |
|  |  | (*c*) Explain how missing data were addressed | N/A |
|  |  | (*d*) If applicable, describe analytical methods taking account of sampling strategy | N/A |
|  |  | (*e*) Describe any sensitivity analyses | N/A |
| Results | | | |
| Participants | 13* | (a) Report numbers of individuals at each stage of study—eg numbers potentially eligible, examined for eligibility, confirmed eligible, included in the study, completing follow-up, and analyzed | Provided under Demographic and clinical characteristics of the pharmacists section and Table 1 |
|  |  | (b) Give reasons for non-participation at each stage | N/A |
|  |  | (c) Consider use of a flow diagram | N/A |
| Descriptive data | 14* | (a) Give characteristics of study participants (eg demographic, clinical, social) and information on exposures and potential confounders | Provided under Demographic and clinical characteristics of the pharmacists section and Table 1 |
|  |  | (b) Indicate number of participants with missing data for each variable of interest | N/A |
| Outcome data | 15* | Report numbers of outcome events or summary measures | Provided throughout the results section |
| Main results | 16 | (*a*) Give unadjusted estimates and, if applicable, confounder-adjusted estimates and their precision (eg, 95% confidence interval). Make clear which confounders were adjusted for and why they were included | Provided throughout the results section |
|  |  | (*b*) Report category boundaries when continuous variables were categorized | Provided throughout the results section |
|  |  | (*c*) If relevant, consider translating estimates of relative risk into absolute risk for a meaningful time period | N/A |
| Other analyses | 17 | Report other analyses done—eg analyses of subgroups and interactions, and sensitivity analyses | N/A |
| Discussion | | | |
| Key results | 18 | summarize key results with reference to study objectives | First paragraph of the Discussion section |
| Limitations | 19 | Discuss limitations of the study, taking into account sources of potential bias or imprecision. Discuss both direction and magnitude of any potential bias | Provided under Limitations |
| Interpretation | 20 | Give a cautious overall interpretation of results considering objectives, limitations, multiplicity of analyses, results from similar studies, and other relevant evidence | Provided in the Discussion section |
| Generalizability | 21 | Discuss the generalizability (external validity) of the study results | Discussed in the Discussion section |
| Other information | | | |
| Funding | 22 | Give the source of funding and the role of the funders for the present study and, if applicable, for the original study on which the present article is based | Provided in Declarations |

**Supplementary Table S2:** The questionnaire

**Section 1: In this section, we need to collect your demographic and professional details**

| 1 | Please indicate your gender: |
| --- | --- |
|  | □ Male □ Female |
| 2 | Please provide your age in years: .................... |
| 3 | Please indicate your academic degree in pharmacy: |
|  | □ BSc Pharmacy □ Pharm.D □ MSc in Pharmacy □ PhD in Pharmacy |
| 4 | From which country did you obtain your pharmacy degree? |
|  | □ Palestine □ Other |
| 5 | In which year did you obtain your pharmacy degree? …............ |
| 6 | Since how many years have you been working as a pharmacist in a hospital? ….......... |
| 7 | Where do you currently practice? |
|  | □ Governmental hospital □ Private hospital |

**Section 2: In this section, we need to gather information on the nature of PK courses that you have taken during the pharmacy degree program**

| 1 | **What was the nature of the PK courses that you had taken during your pharmacy degree program?** |
| --- | --- |
|  | □ Basic courses □ Clinical courses □ Both |
| 2 | **How were the PK courses that you had taken during your pharmacy degree program?** |
|  | □ Standalone courses □ Integrated/part of other courses like pharmacotherapy, pharmaceutics, and/or pharmacology |
| 3 | **Have you received continuing education courses related to PK?** |
|  | □ Yes □ No |

**Section 3: On a scale of 1-5 (1 = strongly disagree, 5 = strongly agree), how would you rate the following statements?**

| **#** | **Statement** | **1. Strongly disagree** | **2. Disagree** | **3. Neutral** | **4. Agree** | **5. Strongly agree** |
| --- | --- | --- | --- | --- | --- | --- |
| 1 | The PK courses I received during my pharmacy education were important to my current practice |  |  |  |  |  |
| 2 | The PK courses I received during my pharmacy education were relevant to my current practice |  |  |  |  |  |
| 3 | The PK courses I received during my pharmacy education could have been taught better |  |  |  |  |  |
| 4 | The method used to teach the PK courses during my pharmacy education were effective |  |  |  |  |  |
| 5 | The contents of the PK courses I received during my pharmacy education were adequate |  |  |  |  |  |
| 6 | The depth of the PK courses I received during my pharmacy education was appropriate to prepare me for my future clinical roles |  |  |  |  |  |

**Section 4: On a scale of 1-5 (1 = none of the time, 5 = all/most of the time), how would you rate the following statement?**

| **#** | **Statement** | **1. None of the time** | **2. A little bit of the time** | **3. Some of the time** | **4. A great deal of the time** | **5. All/most of the time** |
| --- | --- | --- | --- | --- | --- | --- |
| 1 | I utilize the PK knowledge gained through my pharmacy education in my current practice |  |  |  |  |  |

**Section 5: On a scale of 1-5 (1 = completely inadequate, 5 = completely adequate), how would you rate the following statement?**

| **#** | **Statement** | **1. Completely inadequate** | **2. Inadequate** | **3. A little inadequate** | **4. Some gaps exist** | **5. Completely adequate** |
| --- | --- | --- | --- | --- | --- | --- |
| 1 | I consider my current PK skills in allowing me to provide optimal patient care adequate |  |  |  |  |  |

**Section 6: On a scale of 1-10 (1 = low difficulty, 10 = high difficulty), how would you rate the following statement?**

| **#** | **Statement** | 1 | 2 | 3 | 4 | 5 | 6 | 7 | 8 | 9 | 10 |
| --- | --- | --- | --- | --- | --- | --- | --- | --- | --- | --- | --- |
| 1 | I think the application of PK knowledge and skills is difficult to implement |  |  |  |  |  |  |  |  |  |  |

**Section 7: On a scale of 1-5 (1 = extremely unimportant, 5 = extremely important), how would you rate the following statements?**

| **#** | **Statement** | **1. Extremely unimportant barrier** | **2. Unimportant barrier** | **3. Neutral** | **4. Important barrier** | **5. Extremely important barrier** |
| --- | --- | --- | --- | --- | --- | --- |
| 1 | Lack of practical knowledge |  |  |  |  |  |
| 2 | Lack of continuing education relevant to PK |  |  |  |  |  |
| 3 | Lack of role model at work place who knows and applies PK |  |  |  |  |  |
| 4 | Poor understanding of PK by the health care professionals other than pharmacists |  |  |  |  |  |
| 5 | Poor understanding of PK by pharmacists |  |  |  |  |  |

BSc: Bachelor of Science, MSc: Master of Science, Pharm.D: Doctor of Pharmacy, PhD: Doctor of Philosophy, PK: Pharmacokinetics

**Supplementary Table S3:** Countries from where the pharmacy degree was obtained

| **Country** | **n** | **%** |
| --- | --- | --- |
| Palestine | 104 | 71.7 |
| Jordan | 23 | 15.9 |
| The Russian Federation | 4 | 2.8 |
| Italy | 3 | 2.1 |
| Syria | 2 | 1.4 |
| Iraq | 1 | 0.7 |
| Greece | 1 | 0.7 |
| France | 1 | 0.7 |
| Egypt | 1 | 0.7 |
| United Arab Emirates | 1 | 0.7 |
| The Philippines | 1 | 0.7 |
| Australia | 1 | 0.7 |
| Pakistan | 1 | 0.7 |
| Turkey | 1 | 0.7 |

**Supplementary Table S4:** Detailed scores

|  | **Characteristic** |  |  |  |
| --- | --- | --- | --- | --- |
| **Item #** | **Gender** | **Q1** | **Median** | **Q2** |
| I01 | Male | 4.0 | 4.0 | 4.0 |
|  | Female | 3.0 | 4.0 | 4.0 |
| I02 | Male | 3.0 | 4.0 | 4.0 |
|  | Female | 3.0 | 4.0 | 4.0 |
| I03 | Male | 3.0 | 4.0 | 4.0 |
|  | Female | 3.0 | 4.0 | 4.0 |
| I04 | Male | 3.0 | 4.0 | 4.0 |
|  | Female | 3.0 | 3.0 | 4.0 |
| I05 | Male | 3.0 | 3.0 | 4.0 |
|  | Female | 2.0 | 3.0 | 4.0 |
| I06 | Male | 3.0 | 3.0 | 4.0 |
|  | Female | 2.0 | 3.0 | 4.0 |
| I07 | Male | 2.5 | 3.0 | 3.5 |
|  | Female | 2.0 | 3.0 | 3.0 |
| I08 | Male | 3.5 | 4.0 | 5.0 |
|  | Female | 3.0 | 4.0 | 5.0 |
| I09 | Male | 4.5 | 6.0 | 8.0 |
|  | Female | 4.0 | 5.0 | 7.0 |
| I10 | Male | 3.0 | 4.0 | 4.0 |
|  | Female | 3.0 | 4.0 | 4.0 |
| I11 | Male | 3.0 | 4.0 | 4.0 |
|  | Female | 4.0 | 4.0 | 4.0 |
| I12 | Male | 3.0 | 4.0 | 4.0 |
|  | Female | 3.0 | 4.0 | 4.0 |
| I13 | Male | 3.0 | 4.0 | 4.0 |
|  | Female | 3.0 | 4.0 | 5.0 |
| I14 | Male | 3.0 | 4.0 | 4.0 |
|  | Female | 3.0 | 4.0 | 4.0 |
| **Item** | **Age (years)** | **Q1** | **Median** | **Q2** |
| I01 | < 40 | 3.0 | 4.0 | 4.0 |
|  | ≥ 40 | 3.0 | 4.0 | 4.0 |
| I02 | < 40 | 3.0 | 4.0 | 4.0 |
|  | ≥ 40 | 3.0 | 4.0 | 4.0 |
| I03 | < 40 | 3.0 | 4.0 | 4.0 |
|  | ≥ 40 | 3.0 | 3.5 | 4.0 |
| I04 | < 40 | 3.0 | 3.0 | 4.0 |
|  | ≥ 40 | 3.0 | 4.0 | 4.0 |
| I05 | < 40 | 2.0 | 3.0 | 4.0 |
|  | ≥ 40 | 3.0 | 4.0 | 4.0 |
| I06 | < 40 | 2.0 | 3.0 | 4.0 |
|  | ≥ 40 | 3.0 | 4.0 | 4.0 |
| I07 | < 40 | 2.0 | 3.0 | 3.0 |
|  | ≥ 40 | 2.0 | 3.0 | 3.0 |
| I08 | < 40 | 3.0 | 4.0 | 5.0 |
|  | ≥ 40 | 3.0 | 4.0 | 5.0 |
| I09 | < 40 | 4.0 | 5.0 | 7.0 |
|  | ≥ 40 | 5.0 | 5.5 | 8.0 |
| I10 | < 40 | 3.0 | 4.0 | 4.0 |
|  | ≥ 40 | 3.0 | 4.0 | 4.0 |
| I11 | < 40 | 4.0 | 4.0 | 4.0 |
|  | ≥ 40 | 4.0 | 4.0 | 5.0 |
| I12 | < 40 | 3.0 | 4.0 | 4.0 |
|  | ≥ 40 | 3.0 | 4.0 | 4.0 |
| I13 | < 40 | 3.0 | 4.0 | 5.0 |
|  | ≥ 40 | 3.0 | 4.0 | 4.0 |
| I14 | < 40 | 3.0 | 4.0 | 4.0 |
|  | ≥ 40 | 3.0 | 4.0 | 4.0 |
| **Item** | **Academic degree in pharmacy** | **Q1** | **Median** | **Q2** |
| I01 | BSc Pharmacy | 3.0 | 4.0 | 4.0 |
|  | Pharm.D | 3.0 | 4.0 | 4.0 |
|  | MSc/PhD in Pharmacy | 4.0 | 4.0 | 5.0 |
| I02 | BSc Pharmacy | 3.0 | 4.0 | 4.0 |
|  | Pharm.D | 3.0 | 4.0 | 4.0 |
|  | MSc/PhD in Pharmacy | 3.0 | 4.0 | 4.0 |
| I03 | BSc Pharmacy | 3.0 | 4.0 | 4.0 |
|  | Pharm.D | 3.0 | 4.0 | 4.0 |
|  | MSc/PhD in Pharmacy | 3.0 | 4.0 | 4.0 |
| I04 | BSc Pharmacy | 3.0 | 3.0 | 4.0 |
|  | Pharm.D | 3.0 | 3.0 | 4.0 |
|  | MSc/PhD in Pharmacy | 3.0 | 3.0 | 4.0 |
| I05 | BSc Pharmacy | 3.0 | 3.0 | 4.0 |
|  | Pharm.D | 3.0 | 3.0 | 4.0 |
|  | MSc/PhD in Pharmacy | 2.0 | 4.0 | 4.0 |
| I06 | BSc Pharmacy | 2.5 | 3.0 | 4.0 |
|  | Pharm.D | 2.0 | 3.0 | 4.0 |
|  | MSc/PhD in Pharmacy | 3.0 | 3.0 | 4.0 |
| I07 | BSc Pharmacy | 2.0 | 3.0 | 3.0 |
|  | Pharm.D | 2.0 | 3.0 | 3.0 |
|  | MSc/PhD in Pharmacy | 3.0 | 3.0 | 3.0 |
| I08 | BSc Pharmacy | 3.0 | 4.0 | 5.0 |
|  | Pharm.D | 3.0 | 4.0 | 5.0 |
|  | MSc/PhD in Pharmacy | 4.0 | 4.0 | 5.0 |
| I09 | BSc Pharmacy | 5.0 | 5.0 | 7.0 |
|  | Pharm.D | 4.0 | 5.0 | 6.5 |
|  | MSc/PhD in Pharmacy | 3.0 | 5.0 | 6.0 |
| I10 | BSc Pharmacy | 3.0 | 4.0 | 4.0 |
|  | Pharm.D | 4.0 | 4.0 | 5.0 |
|  | MSc/PhD in Pharmacy | 3.0 | 4.0 | 4.0 |
| I11 | BSc Pharmacy | 3.0 | 4.0 | 4.0 |
|  | Pharm.D | 4.0 | 4.0 | 4.0 |
|  | MSc/PhD in Pharmacy | 4.0 | 4.0 | 5.0 |
| I12 | BSc Pharmacy | 3.0 | 4.0 | 4.0 |
|  | Pharm.D | 4.0 | 4.0 | 4.5 |
|  | MSc/PhD in Pharmacy | 3.0 | 3.0 | 4.0 |
| I13 | BSc Pharmacy | 3.0 | 4.0 | 4.0 |
|  | Pharm.D | 4.0 | 4.0 | 5.0 |
|  | MSc/PhD in Pharmacy | 3.0 | 4.0 | 5.0 |
| I14 | BSc Pharmacy | 3.0 | 4.0 | 4.0 |
|  | Pharm.D | 4.0 | 4.0 | 4.0 |
|  | MSc/PhD in Pharmacy | 3.0 | 4.0 | 5.0 |
| **Item** | **Country from where the pharmacy degree was obtained** | **Q1** | **Median** | **Q2** |
| I01 | Palestine | 3.0 | 4.0 | 4.0 |
|  | Others | 3.0 | 4.0 | 4.0 |
| I02 | Palestine | 3.0 | 4.0 | 4.0 |
|  | Others | 3.0 | 4.0 | 4.0 |
| I03 | Palestine | 3.0 | 4.0 | 4.0 |
|  | Others | 3.0 | 3.0 | 4.0 |
| I04 | Palestine | 3.0 | 3.0 | 4.0 |
|  | Others | 3.0 | 4.0 | 4.0 |
| I05 | Palestine | 2.0 | 3.0 | 4.0 |
|  | Others | 3.0 | 4.0 | 4.0 |
| I06 | Palestine | 2.0 | 3.0 | 4.0 |
|  | Others | 3.0 | 4.0 | 4.0 |
| I07 | Palestine | 2.0 | 3.0 | 3.0 |
|  | Others | 2.0 | 3.0 | 3.0 |
| I08 | Palestine | 3.0 | 4.0 | 5.0 |
|  | Others | 4.0 | 5.0 | 5.0 |
| I09 | Palestine | 4.0 | 5.0 | 7.0 |
|  | Others | 5.0 | 6.0 | 8.0 |
| I10 | Palestine | 4.0 | 4.0 | 5.0 |
|  | Others | 3.0 | 4.0 | 4.0 |
| I11 | Palestine | 4.0 | 4.0 | 4.0 |
|  | Others | 3.0 | 4.0 | 4.0 |
| I12 | Palestine | 3.0 | 4.0 | 4.0 |
|  | Others | 3.0 | 4.0 | 4.0 |
| I13 | Palestine | 3.0 | 4.0 | 5.0 |
|  | Others | 3.0 | 4.0 | 4.0 |
| I14 | Palestine | 3.0 | 4.0 | 4.5 |
|  | Others | 3.0 | 4.0 | 4.0 |
| **Item** | **Time elapsed since the pharmacy degree was obtained (years)** | **Q1** | **Median** | **Q2** |
| I01 | < 10 | 3.0 | 4.0 | 4.0 |
|  | ≥ 10 | 3.0 | 4.0 | 4.0 |
| I02 | < 10 | 3.0 | 4.0 | 4.0 |
|  | ≥ 10 | 3.0 | 4.0 | 4.0 |
| I03 | < 10 | 3.0 | 4.0 | 4.0 |
|  | ≥ 10 | 3.0 | 4.0 | 4.0 |
| I04 | < 10 | 3.0 | 3.0 | 4.0 |
|  | ≥ 10 | 3.0 | 3.0 | 4.0 |
| I05 | < 10 | 2.0 | 3.0 | 4.0 |
|  | ≥ 10 | 3.0 | 3.0 | 4.0 |
| I06 | < 10 | 2.0 | 3.0 | 4.0 |
|  | ≥ 10 | 3.0 | 3.0 | 4.0 |
| I07 | < 10 | 2.0 | 3.0 | 3.0 |
|  | ≥ 10 | 2.0 | 3.0 | 3.0 |
| I08 | < 10 | 3.0 | 4.0 | 5.0 |
|  | ≥ 10 | 3.0 | 4.0 | 5.0 |
| I09 | < 10 | 4.0 | 5.0 | 7.0 |
|  | ≥ 10 | 5.0 | 6.0 | 7.5 |
| I10 | < 10 | 3.0 | 4.0 | 5.0 |
|  | ≥ 10 | 3.0 | 4.0 | 4.0 |
| I11 | < 10 | 4.0 | 4.0 | 4.0 |
|  | ≥ 10 | 3.0 | 4.0 | 5.0 |
| I12 | < 10 | 3.0 | 4.0 | 4.0 |
|  | ≥ 10 | 3.0 | 4.0 | 4.0 |
| I13 | < 10 | 3.0 | 4.0 | 5.0 |
|  | ≥ 10 | 3.0 | 4.0 | 4.0 |
| I14 | < 10 | 3.0 | 4.0 | 4.0 |
|  | ≥ 10 | 3.0 | 4.0 | 4.0 |
| **Item** | **Length of working experience as a pharmacist in a hospital (years)** | **Q1** | **Median** | **Q2** |
| I01 | < 5 | 3.0 | 4.0 | 4.0 |
|  | ≥ 5 | 3.0 | 4.0 | 4.0 |
| I02 | < 5 | 3.0 | 4.0 | 4.0 |
|  | ≥ 5 | 3.0 | 4.0 | 4.0 |
| I03 | < 5 | 3.0 | 4.0 | 4.0 |
|  | ≥ 5 | 3.0 | 4.0 | 4.0 |
| I04 | < 5 | 3.0 | 3.0 | 4.0 |
|  | ≥ 5 | 3.0 | 3.0 | 4.0 |
| I05 | < 5 | 3.0 | 3.0 | 4.0 |
|  | ≥ 5 | 2.0 | 3.0 | 4.0 |
| I06 | < 5 | 2.0 | 3.0 | 4.0 |
|  | ≥ 5 | 2.0 | 3.0 | 4.0 |
| I07 | < 5 | 2.0 | 3.0 | 3.0 |
|  | ≥ 5 | 2.0 | 3.0 | 3.0 |
| I08 | < 5 | 3.0 | 4.0 | 5.0 |
|  | ≥ 5 | 3.0 | 4.0 | 5.0 |
| I09 | < 5 | 4.0 | 5.0 | 7.0 |
|  | ≥ 5 | 5.0 | 5.0 | 7.0 |
| I10 | < 5 | 4.0 | 4.0 | 5.0 |
|  | ≥ 5 | 3.0 | 4.0 | 4.0 |
| I11 | < 5 | 4.0 | 4.0 | 4.0 |
|  | ≥ 5 | 3.0 | 4.0 | 5.0 |
| I12 | < 5 | 3.0 | 4.0 | 4.0 |
|  | ≥ 5 | 3.0 | 4.0 | 4.0 |
| I13 | < 5 | 3.0 | 4.0 | 5.0 |
|  | ≥ 5 | 3.0 | 4.0 | 4.5 |
| I14 | < 5 | 3.0 | 4.0 | 4.0 |
|  | ≥ 5 | 3.0 | 4.0 | 4.0 |
| **Item** | **Type of hospital** | **Q1** | **Median** | **Q2** |
| I01 | Government | 3.0 | 4.0 | 4.0 |
|  | Private | 3.0 | 4.0 | 4.0 |
| I02 | Government | 3.0 | 4.0 | 4.0 |
|  | Private | 3.0 | 4.0 | 4.0 |
| I03 | Government | 3.0 | 4.0 | 4.0 |
|  | Private | 3.0 | 4.0 | 4.0 |
| I04 | Government | 3.0 | 3.0 | 4.0 |
|  | Private | 3.0 | 3.0 | 4.0 |
| I05 | Government | 2.0 | 3.0 | 4.0 |
|  | Private | 3.0 | 3.0 | 4.0 |
| I06 | Government | 2.0 | 3.0 | 4.0 |
|  | Private | 3.0 | 3.0 | 4.0 |
| I07 | Government | 2.0 | 3.0 | 3.0 |
|  | Private | 2.0 | 3.0 | 3.0 |
| I08 | Government | 3.0 | 4.0 | 5.0 |
|  | Private | 3.0 | 4.0 | 5.0 |
| I09 | Government | 4.0 | 5.0 | 7.0 |
|  | Private | 5.0 | 5.0 | 7.0 |
| I10 | Government | 3.0 | 4.0 | 5.0 |
|  | Private | 3.0 | 4.0 | 4.0 |
| I11 | Government | 3.0 | 4.0 | 4.5 |
|  | Private | 4.0 | 4.0 | 4.0 |
| I12 | Government | 3.0 | 4.0 | 4.0 |
|  | Private | 3.0 | 4.0 | 4.0 |
| I13 | Government | 3.0 | 4.0 | 5.0 |
|  | Private | 3.0 | 4.0 | 4.0 |
| I14 | Government | 3.0 | 4.0 | 5.0 |
|  | Private | 3.0 | 4.0 | 4.0 |
| **Item** | **Nature of PK courses** | **Q1** | **Median** | **Q2** |
| I01 | Basic | 3.0 | 4.0 | 4.0 |
|  | Clinical | 3.0 | 4.0 | 5.0 |
|  | Both | 3.0 | 4.0 | 4.0 |
| I02 | Basic | 3.0 | 4.0 | 4.0 |
|  | Clinical | 3.0 | 4.0 | 4.0 |
|  | Both | 3.0 | 4.0 | 4.0 |
| I03 | Basic | 3.0 | 4.0 | 4.0 |
|  | Clinical | 3.0 | 3.0 | 4.0 |
|  | Both | 3.0 | 4.0 | 4.0 |
| I04 | Basic | 3.0 | 3.0 | 4.0 |
|  | Clinical | 3.0 | 4.0 | 4.0 |
|  | Both | 3.0 | 3.0 | 4.0 |
| I05 | Basic | 2.0 | 3.0 | 4.0 |
|  | Clinical | 4.0 | 4.0 | 4.0 |
|  | Both | 3.0 | 3.0 | 4.0 |
| I06 | Basic | 2.0 | 3.0 | 4.0 |
|  | Clinical | 3.0 | 3.0 | 4.0 |
|  | Both | 3.0 | 3.0 | 4.0 |
| I07 | Basic | 2.0 | 3.0 | 3.0 |
|  | Clinical | 2.0 | 3.0 | 3.0 |
|  | Both | 2.0 | 3.0 | 3.5 |
| I08 | Basic | 3.0 | 4.0 | 5.0 |
|  | Clinical | 3.0 | 4.0 | 5.0 |
|  | Both | 4.0 | 4.0 | 5.0 |
| I09 | Basic | 5.0 | 6.0 | 7.5 |
|  | Clinical | 3.0 | 5.0 | 5.0 |
|  | Both | 4.0 | 5.0 | 6.0 |
| I10 | Basic | 3.0 | 4.0 | 5.0 |
|  | Clinical | 3.0 | 4.0 | 4.0 |
|  | Both | 3.0 | 4.0 | 4.0 |
| I11 | Basic | 3.0 | 4.0 | 4.0 |
|  | Clinical | 4.0 | 4.0 | 4.0 |
|  | Both | 4.0 | 4.0 | 4.0 |
| I12 | Basic | 3.0 | 4.0 | 4.5 |
|  | Clinical | 3.0 | 4.0 | 4.0 |
|  | Both | 3.0 | 4.0 | 4.0 |
| I13 | Basic | 3.0 | 4.0 | 4.5 |
|  | Clinical | 4.0 | 4.0 | 5.0 |
|  | Both | 3.0 | 4.0 | 5.0 |
| I14 | Basic | 3.0 | 4.0 | 4.0 |
|  | Clinical | 3.0 | 4.0 | 4.0 |
|  | Both | 3.0 | 4.0 | 4.0 |
| **Item** | **How PK courses were taught** | **Q1** | **Median** | **Q2** |
| I01 | Standalone | 3.0 | 4.0 | 5.0 |
|  | Integrated | 3.0 | 4.0 | 4.0 |
| I02 | Standalone | 3.0 | 4.0 | 4.0 |
|  | Integrated | 3.0 | 4.0 | 4.0 |
| I03 | Standalone | 3.0 | 4.0 | 4.0 |
|  | Integrated | 3.0 | 4.0 | 4.0 |
| I04 | Standalone | 3.0 | 3.0 | 4.0 |
|  | Integrated | 3.0 | 3.0 | 4.0 |
| I05 | Standalone | 2.0 | 3.0 | 4.0 |
|  | Integrated | 3.0 | 3.0 | 4.0 |
| I06 | Standalone | 2.0 | 3.0 | 4.0 |
|  | Integrated | 3.0 | 3.0 | 4.0 |
| I07 | Standalone | 2.0 | 3.0 | 3.0 |
|  | Integrated | 2.0 | 3.0 | 3.0 |
| I08 | Standalone | 3.0 | 4.0 | 5.0 |
|  | Integrated | 4.0 | 4.0 | 5.0 |
| I09 | Standalone | 4.0 | 5.0 | 7.0 |
|  | Integrated | 5.0 | 5.0 | 7.0 |
| I10 | Standalone | 4.0 | 4.0 | 5.0 |
|  | Integrated | 3.0 | 4.0 | 4.0 |
| I11 | Standalone | 4.0 | 4.0 | 4.0 |
|  | Integrated | 3.0 | 4.0 | 4.0 |
| I12 | Standalone | 3.0 | 4.0 | 5.0 |
|  | Integrated | 3.0 | 4.0 | 4.0 |
| I13 | Standalone | 3.0 | 4.0 | 5.0 |
|  | Integrated | 3.0 | 4.0 | 4.5 |
| I14 | Standalone | 4.0 | 4.0 | 4.0 |
|  | Integrated | 3.0 | 4.0 | 4.0 |

BSc: Bachelor of Science, MSc: Master of Science, Pharm.D: Doctor of Pharmacy, PhD: Doctor of Philosophy, PK: Pharmacokinetics, Q1: Lower quartile, Q3: Upper quartile, I01: The PK courses I received during my pharmacy education were important to my current practice, I02: The PK courses I received during my pharmacy education were relevant to my current practice, I03: The PK courses I received during my pharmacy education could have been taught in a better way, I04: The method used to teach the PK courses during my pharmacy education were effective, I05: The contents of the PK courses I received during my pharmacy education were adequate, I06: The depth of the PK courses I received during my pharmacy education was appropriate to prepare me for my future clinical roles, I07: I utilize the PK knowledge gained through my pharmacy education in my current practice, I08: I consider my current PK skills in allowing me to provide optimal patient care adequate, I09: I think PK knowledge and skills are difficult to implement, I10: Lack of practical knowledge, I11: Lack of continuing education relevant to PK, I12: Lack of role model at work place who knows and applies PK, I13: Poor understanding of PK by the health care professionals other than pharmacists, I14: Poor understanding of PK by pharmacists
